# Explainable Siamese Neural Networks for Detection of High Fall Risk Older Adults in the Community Based on Gait Analysis

**DOI:** 10.3390/jfmk10010073

**Published:** 2025-02-22

**Authors:** Christos Kokkotis, Kyriakos Apostolidis, Dimitrios Menychtas, Ioannis Kansizoglou, Evangeli Karampina, Maria Karageorgopoulou, Athanasios Gkrekidis, Serafeim Moustakidis, Evangelos Karakasis, Erasmia Giannakou, Maria Michalopoulou, Georgios Ch Sirakoulis, Nikolaos Aggelousis

**Affiliations:** 1 Department of Physical Education and Sport Science, Democritus University of Thrace, 69100 Komotini, Greece; ckokkoti@affil.duth.gr (C.K.); kyapostol@cs.duth.gr (K.A.); dmenychtas@uniwa.gr (D.M.); ekarampi@phyed.duth.gr (E.K.); mkarageo@phyed.duth.gr (M.K.); agkrekid@phyed.duth.gr (A.G.); s.moustakidis@aideas.eu (S.M.); ekarakas@pme.duth.gr (E.K.); egiannak@phyed.duth.gr (E.G.); michal@phyed.duth.gr (M.M.); 2 Laboratory of Robotics and Automation, Department of Production and Management Engineering, Democritus University of Thrace, 67100 Xanthi, Greece; ikansizo@pme.duth.gr; 3 Department of Electrical and Computer Engineering, Democritus University of Thrace, 67100 Xanthi, Greece; gsirak@ee.duth.gr

**Keywords:** falls, biomechanical data, deep learning, explainability

## Abstract

Background/Objectives: Falls among the older adult population represent a significant public health concern, often leading to diminished quality of life and serious injuries that escalate healthcare costs, and they may even prove fatal. Accurate fall risk prediction is therefore crucial for implementing timely preventive measures. However, to date, there is no definitive metric to identify individuals with high risk of experiencing a fall. To address this, the present study proposes a novel approach that transforms biomechanical time-series data, derived from gait analysis, into visual representations to facilitate the application of deep learning (DL) methods for fall risk assessment. Methods: By leveraging convolutional neural networks (CNNs) and Siamese neural networks (SNNs), the proposed framework effectively addresses the challenges of limited datasets and delivers robust predictive capabilities. Results: Through the extraction of distinctive gait-related features and the generation of class-discriminative activation maps using Grad-CAM, the random forest (RF) machine learning (ML) model not only achieves commendable accuracy (83.29%) but also enhances explainability. Conclusions: Ultimately, this study underscores the potential of advanced computational tools and machine learning algorithms to improve fall risk prediction, reduce healthcare burdens, and promote greater independence and well-being among the older adults.

## 1. Introduction

Falls among older adults living in the community represent a critical public health concern, occurring with alarming frequency and carrying significant personal and societal costs [1]. Age-related physical changes, chronic conditions, and environmental factors collectively contribute to these incidents, resulting in a range of outcomes—from minor bruises to severe fractures and head injuries [2]. The repercussions extend beyond the individual patient, increasing the need for medical care, rehabilitation, and general long-term day-to-day assistance. This, in turn, creates expenses that exert a substantial strain on healthcare systems. This underscores the urgency of developing innovative approaches, such as predictive modeling, to identify individuals at heightened risk of falling and take preventative measures. By harnessing data-driven insights and advanced technologies [3]—such as quantitative gait analysis, wearable sensors, and artificial intelligence—healthcare professionals can implement early detection strategies and personalized interventions that improve patient outcomes and reduce overall healthcare costs.

Gait analysis, the systematic examination of an individual’s walking patterns, has long been integral to diagnosing, treating, and managing mobility-related conditions [4]. These include age-related functional declines, neurological disorders, and musculoskeletal injuries. However, conventional gait assessment methods are often time-consuming, costly, and reliant on specialized equipment—factors that limit their widespread adoption. It is also worth mentioning that there are no definitive metrics to assess a person’s balance [5]. Recent developments have begun to address these constraints [6,7,8,9,10]. For example, Mohan et al. have demonstrated the potential of integrating wearable motion sensors with AI [11], while Apostolidis et al. have introduced an innovative visualization approach for biomechanical time-series data in stroke diagnosis using explainable machine learning methods [12]. Collectively, these advancements highlight how modern tools can streamline gait analysis and make it more accessible, informative, and cost-effective.

Convolutional neural networks (CNNs) in particular excel at extracting meaningful patterns from complex visual data, making them well suited for gait analysis tasks. In addition, Siamese neural networks (SNNs) offer a robust means of comparing gait patterns, accommodating subtle variations in appearance and movement that may elude traditional methods. Several recent studies illustrate the diverse applications of SNNs in this domain [13,14,15]. For instance, Zhang et al. [16] proposed a human identification framework that leverages gait energy images and SNNs to more effectively capture gait features, outperforming conventional approaches when tested on large datasets. Similarly, Liu et al. [17] integrated bioinformatics with deep learning-based SNN architectures to refine spatial and temporal gait representations, ultimately enhancing distance metric learning for improved human identification. Beyond identification tasks, Fan et al. [18] demonstrated how SNNs can incorporate inertial sensor data for automatic fall risk assessment in stroke survivors, underscoring the potential clinical impact of these advanced algorithms.

Building on these emerging methodologies, this study has two primary objectives. First, it introduces an innovative tool to visualize biomechanical time-series data for enhanced fall risk prediction. Second, it addresses the challenges posed by limited datasets through the use of SNNs, thereby broadening the utility and precision of gait analysis techniques. By reducing falls and their associated economic burdens, these proactive strategies have the potential to significantly improve quality of life for older adults, fostering independence, well-being, and deeper engagement within the community.

## 2. Materials and Methods

### 2.1. Participants

The participants were individuals aged 55 to 80 of both sexes (male 15% and female 85%) residing in both urban and rural areas of the Eastern Macedonia and Thrace regions in Greece. There were no specific BMI criteria for inclusion; individuals across all BMI categories were eligible to participate. They were recruited through local community centers and social services. Older adults with mobility limitations or cognitive impairments were excluded from the study. To evaluate the occurrence of falls, we utilized the Johns Hopkins Fall Risk Assessment Tool [19]. Our primary focus was on a binary classification task distinguishing between participants at high risk of falling (22 individuals) and those classified as non-fallers (22 individuals), as summarized in Table 1. All participants received a thorough explanation of the study objectives and procedures before providing their written informed consent. This research was approved by the Research Ethics Committee at Democritus University of Thrace (Reference: DUTH/EHDE/28061/165).

### 2.2. Data Collection and Analysis

Upon entering the gait laboratory, participants were given clear instructions regarding the testing process and acclimated to the walking task. Specific anthropometric measurements were taken, and a total of 57 spherical retroreflective markers were meticulously positioned on specific anatomical landmarks across the whole body. These measurements included body mass, height, leg length (measured from the anterior superior iliac spine to the medial malleolus), knee width (mediolateral width across the femoral epicondyles), ankle width (mediolateral distance across the malleoli), and inter-ASIS distance (distance between the left and right anterior superior iliac spines). For the upper body, the measurements included shoulder offset (vertical distance from the center of the glenohumeral joint to the acromion clavicular joint), elbow width (mediolateral width across the medial and lateral epicondyles of the humerus), wrist thickness (distance between the palm and the back side of the wrist), and hand thickness (distance between the dorsal and palmar surfaces of the hand).

The marker set of Conventional Gait Model version 2.4 (CGM 2.4) was used, which was fully integrated with the system’s software (Nexus 2.14). This standardized marker arrangement, as outlined in the existing literature, served as a reference point for subsequent analysis. Subsequently, participants walked a 10 m distance barefoot within the laboratory, maintaining a pace that stayed within ±5% of their individually determined self-selected walking speed (SWS). The SWS was established during a preliminary familiarization session using infrared timing gates and was upheld throughout data collection with the aid of a metronome. Trials continued until a minimum of 10 complete gait cycles were captured for each foot on the force platform. A trial was considered valid only if the foot under observation made clean contact with the force platform and the walking speed remained within ±5% of the individual’s SWS. Data collection involved kinematic measurements recorded at a frequency of 100 Hz through 10 optoelectronic cameras (Vicon T-series, Oxford, UK), and kinetic data were captured at 1000 Hz using two embedded force platforms (Kistler, 9281Β11 and 9281CA, respectively) synchronized with kinematic data.

The subsequent data analysis phase involved the utilization of 5 trials, encompassing complete gait cycles for each subject. Initial contact and toe-off events during the stance phase were identified using the vertical ground reaction force (GRF) with a 20 N threshold. The recorded data and Vicon Nexus software (Nexus 2.14) were employed to determine ipsilateral initial contact.

The kinetic data included ground reaction forces (GRFs), joint moments, joint forces, and center of pressure (CoP). However, only the kinetic data were used in the subsequent analysis to develop a model for detecting high fall risk older adults. This decision was based on the need for an interpretable model that relies on marker-based motion data, which is more practical for real-world applications where force platforms may not be available. In addition, the kinematic features capture key gait characteristics without the complexity of kinetic calculations, making the model more adaptable for clinical and community evaluations.

**Figure 1:**
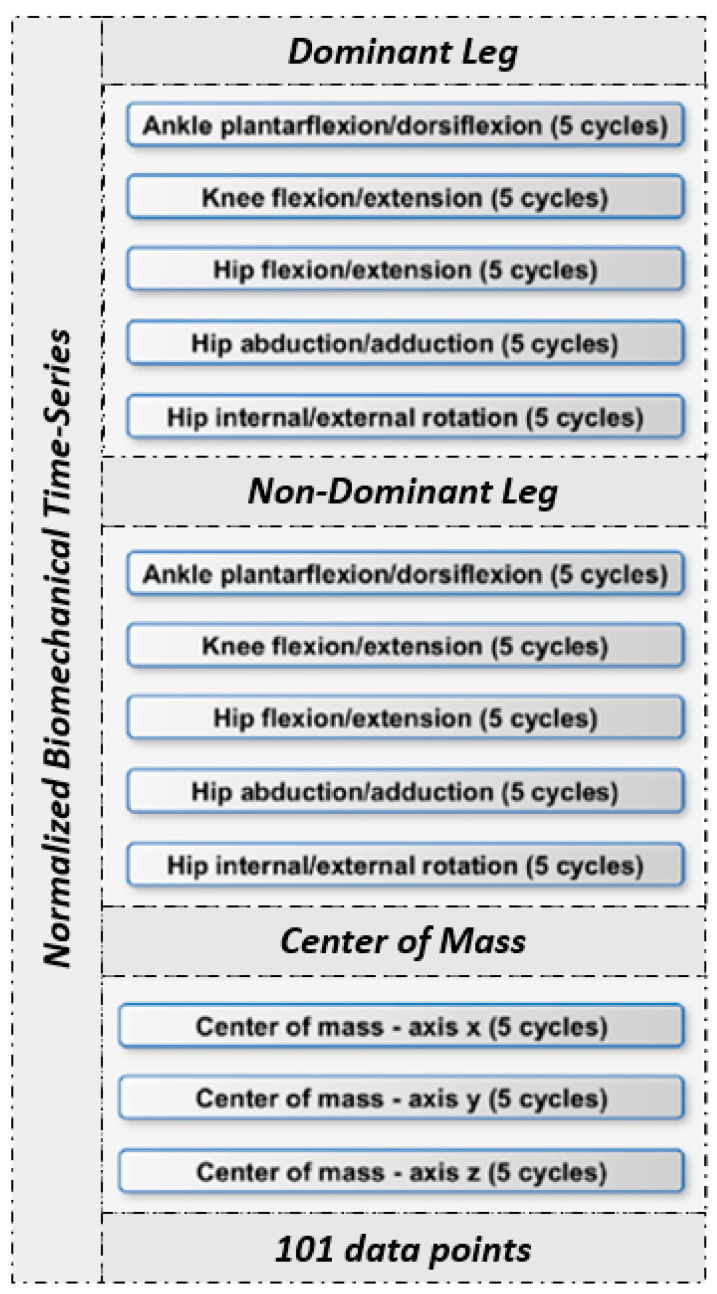
Visual representation of the single 2D matrix per subject [12].

To ensure consistency, kinematic data underwent filtration at 10 Hz using an MSE Woltring filter, while the Conventional Gait Model 2 (CGM2.4) was harnessed to generate the biomechanical time-series (Nexus 2.14, Oxford, UK). From each trial of every subject, specific time-series were extracted. These kinematic datasets encompassed parameters such as ankle plantarflexion/dorsiflexion, knee flexion/extension, hip flexion/extension, hip abduction/adduction, and hip internal and external rotation for both dominant and non-dominant legs, along with three-dimensional center of mass coordinates (Figure 1). Each time-series was standardized to a 101-point scale using cubic spline interpolation. Following proper interpolation of gait cycles, data were normalized to a standardized range, effectively ranging between 0 and 1. This normalization procedure facilitated meaningful cross-subject comparisons and aided in the identification of pertinent differences or trends between distinct groups.

### 2.3. Image Construction

Following the division and standardization of the chosen signals, a two-dimensional (2D) matrix is formulated by combining the respective gait cycles that stem from the same signal, sequenced in a chronological manner. In this matrix, every row is emblematic of a distinct cycle, while each column denotes the temporal progression expressed as a cycle percentage. Within a given row, the i-th element signifies the corresponding percentage duration from the initiation of the cycle, thereby offering a temporal reference for tracking motion evolution. This matrix affords a precise point-to-point alignment of cycles, proving especially valuable for comparative analysis and the discernment of potential patterns or distinctions among subjects. Subsequently, all of these matrices are vertically integrated, culminating in a single 2D matrix per subject. The numerical values within this matrix are confined to the range [0, 1], effectively encapsulating the comprehensive representation of motion patterns in normalized terms.

### 2.4. Learning Methodology

**Figure 2:**
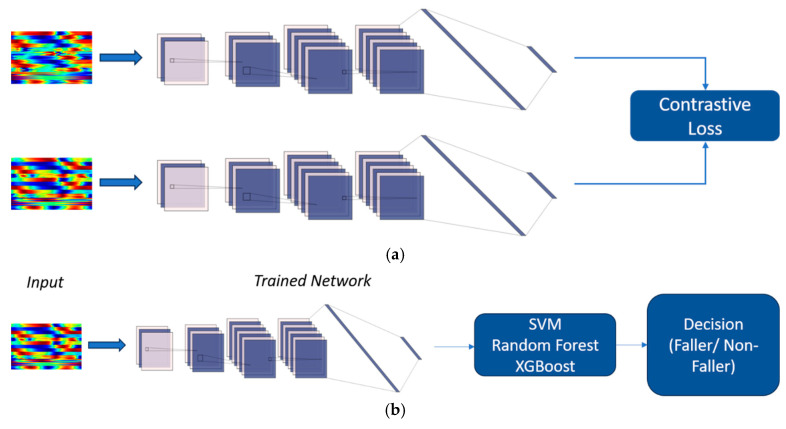
Proposed workflow pipeline for the prediction of high fall risk older adults in the community. (**a**) The concept of the Siamese neural network. (**b**) The proposed pipeline for classifying the images.

One popular type of neural network architecture is the SNN, which is often used when working with limited data [20]. SNNs were originally introduced by Bromley and LeCun for signature verification, and they have since been used in a variety of applications, including face recognition and signature verification. The architecture of an SNN typically involves two identical neural networks that share the same weights and structure (Figure 2). The model is then trained using a similarity function that measures the distance between the feature vectors of two images. We utilized the contrastive loss function for training, which is distance-based, seeks to minimize the Euclidean distance between similar feature vectors, and is described as follows:

(1) $$L=\left(1-Y\right)\frac{1}{2}{\left(Dw\right)}^{2}+\frac{1}{2}{\left\{max(0,m-Dw)\right\}}^{2},$$

The similarity between two vectors is represented by Y, where Y equals 0 if the vectors are similar and 1 if they are dissimilar. Dw denotes the Euclidean distance between the vectors. To evaluate the performance of our model, we utilized the accuracy metric, which is defined as follows.

(2) $$\frac{TP+TN}{TP+TN+FP+FN},$$

where TP are true positive instances, TN are true negative instances, FP are false positive instances, and FN are false negative instances.

In our experiments, we employed a small model consisting of three convolutional blocks, with each block comprising a convolutional layer, a dropout layer, and a batch normalization layer. This model ultimately yields a feature vector of size 5 for each input image. Following training, feature vectors corresponding to images of the same class should have a minimum Euclidean distance between them.

We utilized support vector machines (SVMs) [21], random forest (RF) [22] and eXtreme gradient boosting (XGBoost) [6] machine learning algorithms to classify images based on the feature vectors generated by the SNN.

The explainability of the proposed method was also examined by applying Gradient-weighted Class Activation Mapping (Grad-CAM) to the test images [23]. In the context of medical problems, DL models must be explainable to ensure trust and acceptance, address legal and ethical concerns, detect errors and failures, improve performance, and maintain patient safety. It is therefore imperative that the decision-making process of these DL models be understood by medical professionals, enabling them to make informed decisions for their patients.

Grad-CAM is employed as an explainability tool to elucidate the decision-making process of convolutional neural networks (CNNs) in computer vision tasks. By highlighting the regions within an input image that exerted the greatest influence on CNN’s final prediction, Grad-CAM supports a clearer understanding of why a particular decision was reached. The resultant heatmaps assist researchers and developers in identifying and correcting errors, refining model performance, and fostering trust and transparency in the model’s outputs.

To compute Grad-CAM for a given input image, the following steps are typically performed:

- The input image is processed by the CNN, generating output feature maps;
- The gradient of the predicted class score with respect to the feature maps of the last convolutional layer is calculated;
- The gradients from step 2 are averaged globally to produce a set of weights, which represent the relative importance of each feature map in the final prediction;
- The feature maps of the final convolutional layer are linearly combined using the computed weights, producing the class activation map;
- The class activation map is then passed through a rectified linear unit (ReLU) function. Negative values are set to zero, while positive values remain unchanged. This thresholding generates a heatmap that emphasizes the regions most critical for the predicted class.

### 2.5. Validation Strategy

The dataset utilized in this study consisted of 44 images in total: 22 images represented fallers, while another 22 represented individuals without a history of falls. To ensure a fair and unbiased assessment, a stochastic validation strategy was employed, involving multiple random splits with 70% of the data allocated to training and 30% allocated to testing. Specifically, a 100-time random splitting process was applied to achieve a robust and representative evaluation. As standard practice dictates, the test set exclusively contained previously unseen subjects not included in the training set.

## 3. Results

To assess the effectiveness of the proposed SNN-based architecture, training was conducted using the ADAM optimizer with a learning rate of 0.0005 for 10 epochs, resulting in a loss value of 0.06. The dataset comprised a total of 44 images, evenly split between high-risk fallers (22 images) and non-fallers (22 images). The test subset included six images representing fallers and five images representing non-fallers.

Following the SNN model being trained, feature vectors were extracted and subsequently used to train SVM, RF, and XGBoost models. For all models, SVM, RF, and XGBoost, the training dataset was further divided into 70% training and 30% testing splits. The SVM was configured with an RBF kernel and a regularization parameter C = 1.0, while the RF model employed 100 decision trees. The XGBoost classifier was configured with n estimators = 100, learning rate = 0.3, and maximum depth = 6. The model was evaluated using the log loss metric.

Table 2 summarizes the classification results for each method. The RF model yielded the best performance, achieving an accuracy of 83.29%. The SVM and XGBoost methods followed closely, with accuracies of 81.71% and 81.57%, respectively. In contrast, the Euclidean distance-based approach performed considerably worse, obtaining an accuracy of only 63.60%.

**Figure 3:**
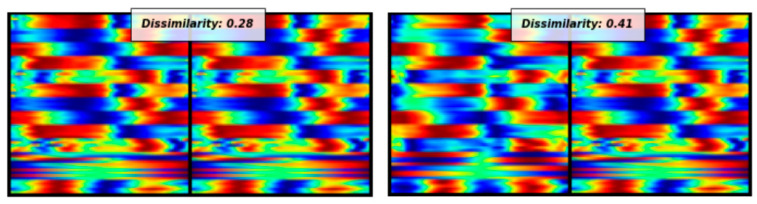
Dissimilarity distance between two non-faller images compared to the dissimilarity distance between a non-faller and a faller image.

An illustration of this concept is provided in Figure 3, which shows that images belonging to the same class exhibit a relatively small dissimilarity distance, while images from different classes display a larger dissimilarity distance.

**Figure 4:**
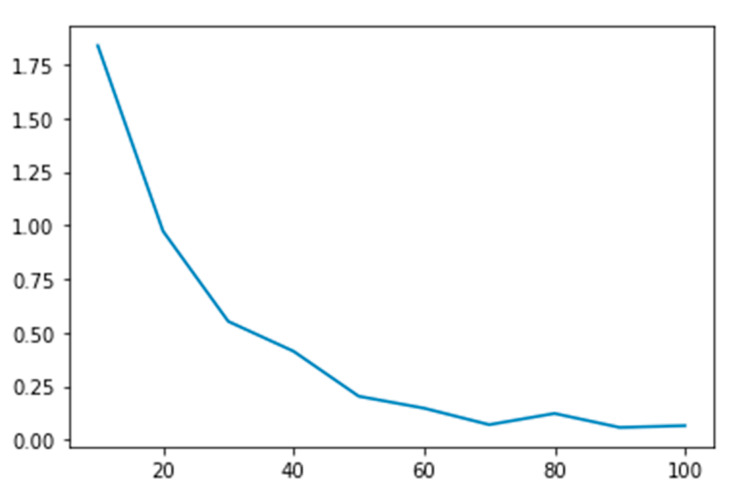
Loss function through iterations during training.

Figure 4 illustrates the loss values as a function of the number of iterations. The observed results indicate that the model stabilizes after approximately 60 iterations, reaching a loss value of about 0.1.

**Figure 5:**
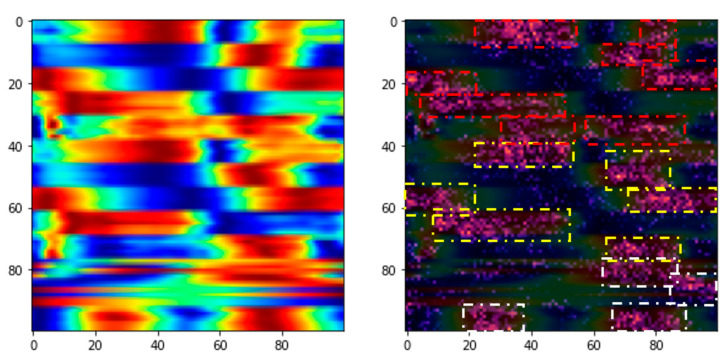
Grad-CAM in a non-faller. Boxes highlight the critical points according to the Grad-CAM algorithm.

**Figure 6:**
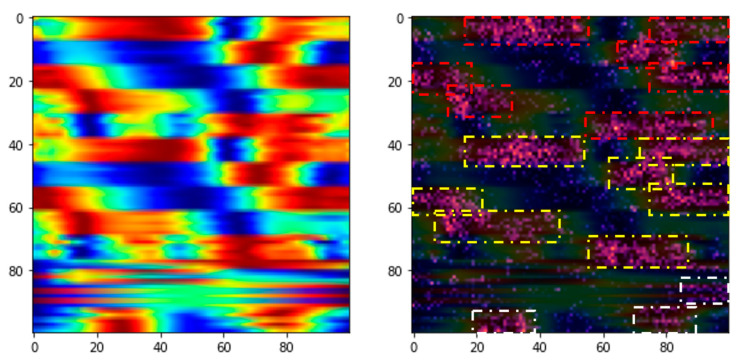
Grad-CAM in a faller. Boxes highlight the critical points according to the Grad-CAM algorithm.

Another noteworthy contribution of this study is the extraction of informative features using the Grad-CAM algorithm. Our diagnostic task demonstrates high performance, achieving an accuracy of 83.29% with the proposed models. Figure 5 and Figure 6 illustrate how Grad-CAM highlights salient image regions for non-faller and faller subjects, respectively, revealing the model’s focus during feature vector generation. As shown, Grad-CAM effectively identifies and emphasizes the most distinct areas between these two groups, primarily concentrated in both legs and at the center of mass, which exhibit the greatest intensity differences.

In the non-faller case (Figure 5), Grad-CAM highlights relatively uniform regions in both legs and the center of mass, indicating well-balanced motion patterns with consistent focus across these key areas. The intensity of the highlighted regions is evenly distributed, suggesting the absence of pronounced asymmetries or irregularities in gait dynamics. Conversely, in the faller case (Figure 6), the Grad-CAM algorithm reveals more localized and uneven critical regions. The highlights in the faller are concentrated around specific segments of the legs and the center of mass, exhibiting noticeable disparities in intensity. These localized regions suggest asymmetrical gait patterns, particularly in one leg, as well as irregularities in center of mass movement. Such patterns could be indicative of instability, reduced coordination, or compensatory mechanisms often observed in fall-prone individuals.

In summary, the comparison between non-faller and faller Grad-CAM visualizations underscores the ability of the algorithm to identify subtle but significant differences in motion dynamics. Specifically, the non-faller shows consistent and balanced intensity highlights across all gait cycle phases, particularly during transitions. In contrast, the faller displays disparities concentrated in the loading response and preswing-to-initial swing phases, where balance and stability tend to break down. The non-faller’s uniform distribution contrasts sharply with the faller’s focalized and imbalanced regions, supporting the model’s capacity to discern critical features relevant to fall risk assessment.

**Figure 7:**
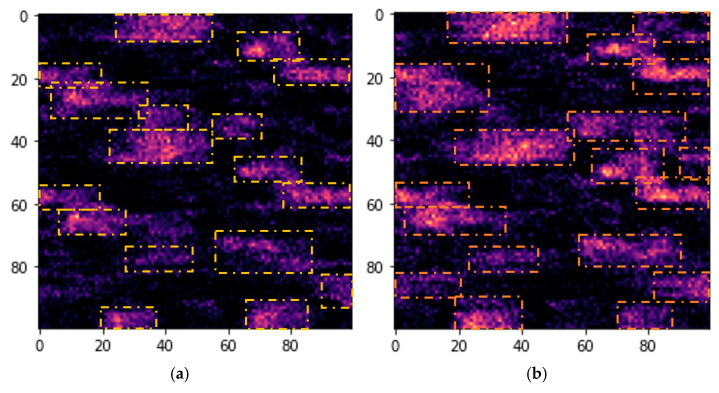
Mean Grad-CAM masks for the testing group of the employed fallers and non-fallers. Specifically, (**a**) presents non-faller participants and (**b**) presents fallers.

To verify the proper functioning of the proposed algorithm, a cumulative mask was created by summing all non-faller Grad-CAM masks, and another cumulative mask was constructed using all faller Grad-CAM masks. Through this approach, it was examined whether the SNN consistently focused on the same regions when classifying new samples. In Figure 7a,b, these resulting masks are presented, thereby demonstrating that the model’s attention remains concentrated within these regions and confirming its consistency in decision-making.

## 4. Discussion

Identifying the risk of falling between older adults based solely on gait analysis remains a formidable challenge in geriatric health assessment. The present study introduces an innovative tool that visually represents biomechanical time-series data, thereby enhancing the predictive accuracy of fall risk evaluation. Furthermore, this work addresses the complexities associated with limited datasets by capitalizing on the capabilities of SNNs. Through this novel approach, the horizons of gait analysis are broadened, providing more comprehensive insights into fall-related dynamics and outcomes.

Previous research has demonstrated that SNNs exhibit considerable effectiveness in scenarios constrained by limited data [24,25]. In this study, given the inherent constraints on participant numbers, biomechanical signals were transformed into visual representations to facilitate SNN training. Despite the modest dataset, the proposed method achieved a promising accuracy rate of 83.29%. This result underscores the potential of image-based representations and SNNs in improving fall risk prediction under data-scarce conditions.

The analysis of Grad-CAM activation regions reveals key biomechanical distinctions between non-fallers and fallers during the gait cycle [23]. For non-fallers, activation patterns appear evenly distributed in both the stance and swing phases, reflecting well-coordinated and stable motion. This uniformity highlights effective neuromuscular control, ensuring smooth transitions between phases. In particular, during critical transitions, such as the preswing and initial swing phases—where the foot is lifted off the ground and propulsion begins—there is a modest, localized increase in activation intensity. This might suggest precise timing and coordination of muscle contractions, particularly in the plantar, hip flexors, and dorsiflexors, which are essential for forward momentum and limb propulsion [26,27,28]. Such activation patterns may also suggest efficient load transfer, joint alignment, and balance, allowing non-fallers to maintain stable gait mechanics throughout the cycle.

On the contrary, those with a high risk of falling exhibit uneven and localized patterns of activation, which signal underlying biomechanical inefficiencies and compensatory adaptations. During the loading response phase—a critical period for weight acceptance after initial contact—activation regions show pronounced asymmetries. This asymmetry suggests a reduced ability to distribute weight evenly between the limbs, possibly due to deficiencies in joint stability, proprioception, or muscle strength [29,30]. The uneven activation likely reflects reliance on compensatory strategies, such as favoring one limb or changing the trajectory of the center of mass, to maintain stability during this phase.

The preswing-to-initial swing transition—another key phase for propulsion and limb advancement—also displays heightened and uneven activation in fallers. This imbalance often involves excessive activation in the muscles of the leading leg. These patterns suggest difficulty in generating sufficient push-off force or controlling the swing trajectory of the advancing limb. Biomechanically, this phase demands precise timing and coordination of ankle plantarflexion, hip flexion, and knee extension, along with stabilization of the pelvis to ensure effective limb clearance and forward motion [31]. The uneven activation in fallers indicates disruptions in these processes, likely stemming from deficits in strength, coordination, or motor control [32,33].

Moreover, the increased engagement of the center of mass along the vertical axis in fallers underscores their difficulty in maintaining dynamic stability. Effective gait requires the controlled oscillation of the center of mass within a narrow range to minimize energy expenditure and preserve momentum [26,34,35]. In fallers, the exaggerated vertical motion likely reflects an effort to compensate for instability, which can disrupt the smooth progression of the gait cycle. This instability becomes particularly critical during the swing phase, where precise neuromuscular control is essential to position the foot for the subsequent stance phase [26].

These findings collectively highlight the sensitivity of specific gait phases—particularly the swing phase and transitions between stance and swing phases—to deficits in motor control, strength, and stability [29,32]. In fallers, the activation patterns suggest a reliance on maladaptive strategies to address these deficits, such as over-recruitment of certain muscle groups or altered movement trajectories. These compensatory mechanisms may temporarily mitigate instability but can exacerbate inefficiencies and increase the risk of falls over time [33]. In contrast, the consistent and balanced activation patterns in non-fallers underline their ability to maintain efficient joint alignment, weight distribution, and coordinated muscle activation, all of which are hallmarks of stable and effective gait mechanics [28].

This study’s limitations primarily stem from the relatively small sample size and reliance on data acquired within a single gait laboratory. Consequently, the generalizability and external validity of the proposed model remain to be fully established. Future research endeavors will aim to address these limitations through external validation using datasets from different environments. Additionally, incorporating novel signals from diverse sources—such as inertial measurement units (IMUs)—may enrich the input data and further refine the model’s predictive capabilities [36]. Exploring advanced explainability methodologies, including the integration of Grad-CAM with SHapley Additive exPlanations (SHAP), holds promise for generating deeper interpretative insights and fostering greater confidence in the model’s decision-making processes. Future studies should also consider models that incorporate additional gait-related factors, such as cognitive changes and preferred walking speed, to enhance predictive accuracy [37]. Integrating force platform measurements could provide detailed information on joint moments and powers during critical gait phases, like terminal stance or preswing. Finally, longitudinal studies will be essential to validate the model’s detection and prognostic capabilities over time.

Distinguishing between healthy older adults and those at high risk of falling based solely on gait analysis remains a formidable challenge in geriatric health assessment. The present study introduces an innovative tool that visually represents biomechanical time-series data, thereby enhancing the predictive accuracy of fall risk evaluation. Furthermore, this work addresses the complexities associated with limited datasets by capitalizing on the capabilities of SNNs. Through this novel approach, the horizons of gait analysis are broadened, providing more comprehensive insights into fall-related dynamics and outcomes.

## 5. Conclusions

In conclusion, this study highlights the multifaceted complexity of understanding human gait and accurately predicting fall risk among the older adults. Despite these challenges, there exists an opportunity to reshape fall risk assessment through advanced methodologies. The integration of CNNs, SNNs, and motion capture technologies has the potential to revolutionize gait analysis, leading to more accurate, accessible, and proactive fall prevention strategies. By improving predictive accuracy, healthcare systems may be alleviated of substantial burdens, while the older adults can preserve their independence and enjoy a higher quality of life. The synergy of innovative computational approaches, enhanced data acquisition methods, and a collective dedication to mitigating fall risks promises to usher in a new era of proactive geriatric healthcare and community well-being.

## Figures and Tables

**Table 1:** Subjects’ characteristics per group (mean ± SD).

	High-Risk Fallers	Non-Fallers
Sex		
Male (%)	4.6	9.2
Female (%)	95.4	90.8
Age (yrs)	70.62 ± 3.93	66.09 ± 5.71
Height (m)	1.54 ± 0.06	1.57 ± 0.09
Weight (Kg)	76.5 ± 21.33	67.37 ± 11.15
BMI (kg/m^2^)	32.43 ± 9.32	27.39 ± 3.33

**Table 2:** Performance on fallers vs. non-fallers classification.

Method	Accuracy (%)	Recall (%)	Precision (%)	F1 Score (%)
Euclidean Distance	63.60	66.70	66.70	66.70
SVM	81.71	81.71	77.82	76.78
RF	83.29	83.29	83.97	80.97
XGBoost	81.57	81.57	80.94	78.99

Abbreviation: SVM: support vector machines; RF: random forest; XGBoost: eXtreme Gradient Boosting.

## Data Availability

The data presented in this study are available on request from the corresponding author. The data are not publicly available due to privacy reasons.
